# Supplementary material for: Evaluation of Dittrichia viscosa Aquaporin Nip1.1 Gene as Marker for Arsenic-Tolerant Plant Selection
Source: Plants (Basel). 2022 Jul 28;11(15):1968. doi: 10.3390/plants11151968 (PMC9370626; doi:10.3390/plants11151968)
Supplement: Supplementary file 1 [file plants-11-01968-s001.zip › plants-1801371-supplementary.pdf]

Supplementary Material

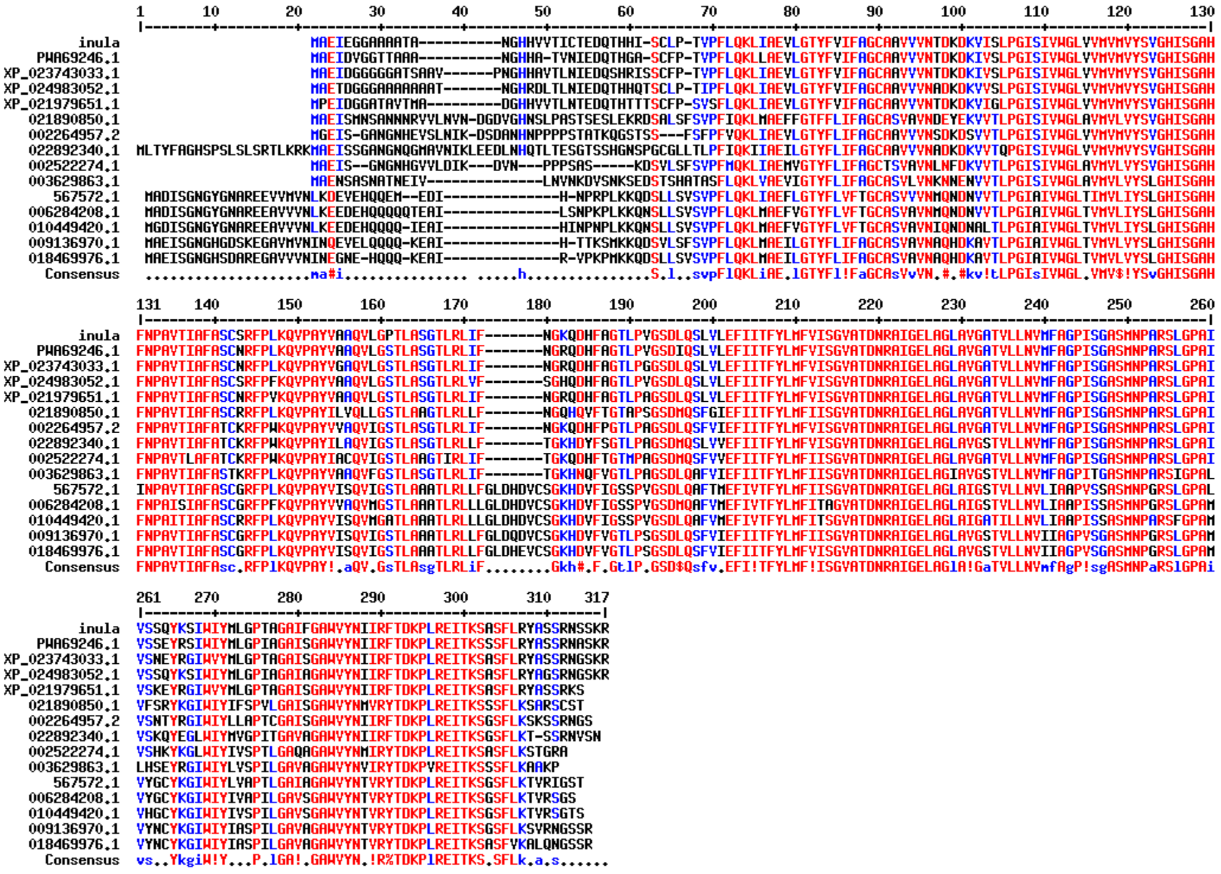

Supplemental figure s1: Alignment of the predicted aminoacid sequence of DvNip1 with members of Arabidopsis and other Nip . Putative Dittrichia viscosa DvNip1 amino acid sequence were aligned using MultiAlign. The identical residues are shaded in red. >inula nip [*Dittrichia viscosa*]; >PWA69246.1 NOD26-like intrinsic protein 1,2 [*Artemisia annua*]; >XP_023743033.1 aquaporin NIP1-2-like [*Lactuca sativa*]; >XP_024983052.1 aquaporin NIP1-1-like [*Cynara cardunculus* var. scolymus]; >XP_021979651.1 aquaporin NIP1-1-like [*Helianthus annuus*]; >567572.1 NOD26-like major intrinsic protein 1 [*Arabidopsis thaliana*]; >006284208.1 aquaporin NIP1-1 [*Capsella rubella*];>010449420.1 PREDICTED: aquaporin NIP1-1 isoform X1 [*Camelina sativa*]; >009136970.1 PREDICTED: aquaporin NIP1-2 [*Brassica rapa*]; >018469976.1 PREDICTED: aquaporin NIP1-2 [*Raphanus sativus*]; >021890850.1 aquaporin NIP1-2 [*Carica papaya*]; >002522274.1 PREDICTED: aquaporin NIP1-2 isoform X1 [*Ricinus communis*]; >002264957.2 PREDICTED: aquaporin NIP1-1 [*Vitis vinifera*]; >003629863.1 nodulin-26 isoform X1 [*Medicago truncatula*]; >022892340.1 aquaporin NIP1-1-like isoform X1 [*Olea europaea* var. sylvestris].

Supplementary Material

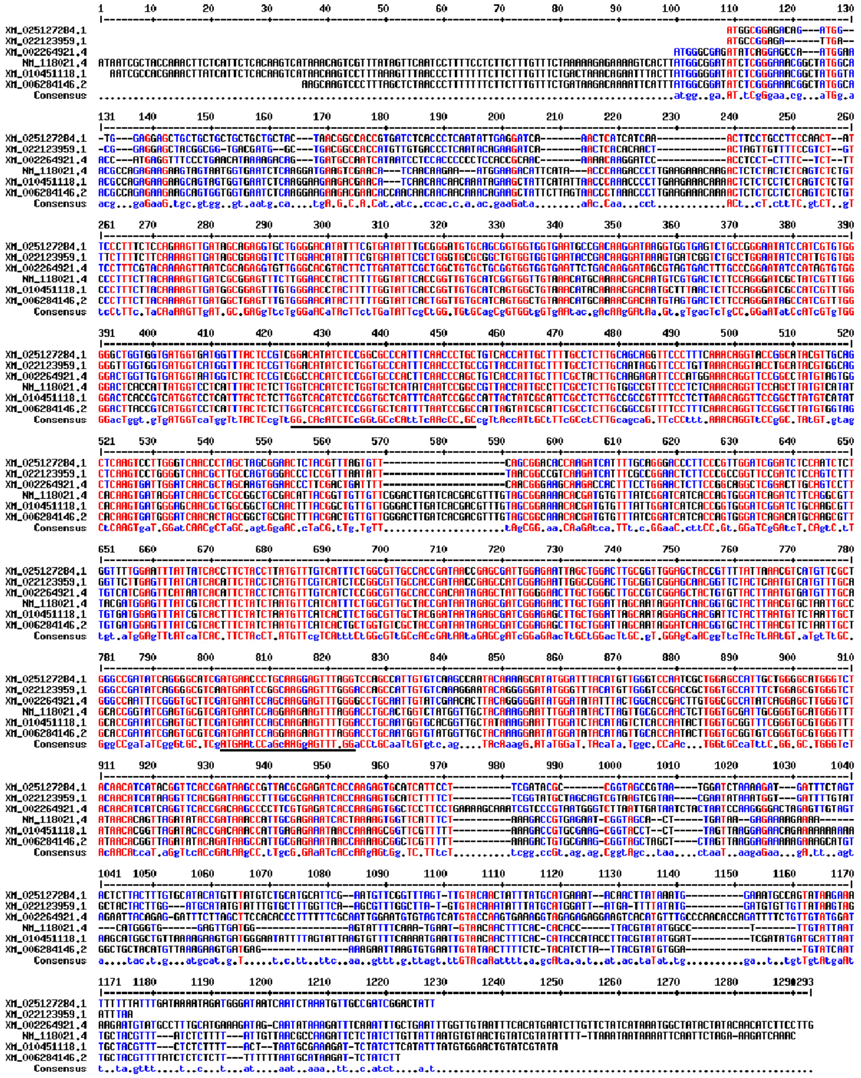

Supplemental figure s2: Nucleotide sequence alignment of Nip1.1 gene. XM_025127284.1 PREDICTED: *Cynara cardunculus* var. scolymus, >XM_022123959.1 PREDICTED: *Helianthus annuus* aquaporin NIP1-1-like , >XM_002264921.4 PREDICTED: *Vitis vinifera* aquaporin NIP1-1, >NM_118021.4 *Arabidopsis thaliana* NOD26-like major intrinsic protein 1 (NLM1), >XM_006284146.2 PREDICTED: *Capsella rubella* aquaporin NIP1-1 were aligned with multiAlign (ref. Multiple sequence alignment with hierarchical clustering" F. CORPET, 1988, Nucl. Acids Res., 16 (22), 10881-10890. The position of degenerate primers was indicated.

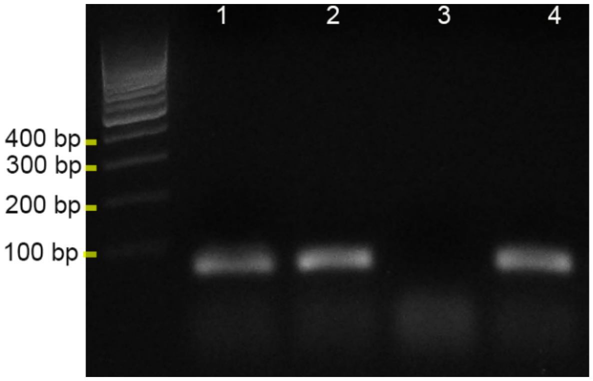

Supplemental Figure s3. Conventional PCR products amplified with each pair of primers revealed a single product of the expected size. Obtained amplimers with AtEF1-A (1), AtAct2 (2), AtAct8 (3) and NtEF1-A (4) primer pairs, visualized on 2.0% agarose gel.

Descriptions | Graphic Summary | Alignments | Taxonomy

Sequences producing significant alignments Download Select columns Show 100

select all 100 sequences selected GenPept Graphics Distance tree of results Multiple alignment MSA Viewer

Description	Scientific Name	Max Score	Total Score	Query Cover	E value	Per. Ident	Acc. Len	Accession
elongation factor 1-alpha [Helianthus annuus]	Helianthus annuus	64.7	64.7	100%	1e-13	100.00%	449	XP_021981523.1
elongation factor 1-alpha-like [Cynara cardunculus var. scolymus]	Cynara cardunculus ...	64.7	64.7	100%	1e-13	100.00%	449	XP_024976594.1
elongation factor 1-alpha [Artemisia annua]	Artemisia annua	64.7	64.7	100%	1e-13	100.00%	449	PWA49076.1
elongation factor 1-alpha [Helianthus annuus]	Helianthus annuus	64.7	64.7	100%	1e-13	100.00%	449	XP_021973407.1
elongation factor 1-alpha-like isoform X1 [Cynara cardunculus var. scolymus]	Cynara cardunculus ...	64.7	64.7	100%	1e-13	100.00%	449	XP_024970426.1
elongation factor 1-alpha isoform X1 [Lactuca sativa]	Lactuca sativa	64.7	64.7	100%	1e-13	100.00%	449	XP_023747111.1
elongation factor 1-alpha-like [Lactuca sativa]	Lactuca sativa	64.7	64.7	100%	1e-13	100.00%	447	XP_042755374.1
hypothetical protein CTI12_AA312610 [Artemisia annua]	Artemisia annua	64.7	64.7	100%	1e-13	100.00%	447	PWA65970.1
elongation factor 1-alpha [Cynara cardunculus var. scolymus]	Cynara cardunculus ...	64.7	64.7	100%	1e-13	100.00%	447	XP_024983118.1
hypothetical protein CTI12_AA312610 [Artemisia annua]	Artemisia annua	64.7	64.7	100%	1e-13	100.00%	447	PWA65971.1
elongation factor 1-alpha 1 [Cynara cardunculus var. scolymus]	Cynara cardunculus ...	64.7	64.7	100%	1e-13	100.00%	447	XP_024985721.1
elongation factor 1-alpha-like [Cynara cardunculus var. scolymus]	Cynara cardunculus ...	64.7	64.7	100%	1e-13	100.00%	447	XP_024968429.1
hypothetical protein CTI12_AA400390 [Artemisia annua]	Artemisia annua	64.7	64.7	100%	1e-13	100.00%	447	PWA58403.1
elongation factor 1-alpha [Artemisia annua]	Artemisia annua	64.7	64.7	100%	1e-13	100.00%	447	PWA48367.1
elongation factor 1-alpha [Helianthus annuus]	Helianthus annuus	64.7	64.7	100%	1e-13	100.00%	447	XP_022006019.1
hypothetical protein Ccrd_021776 [Cynara cardunculus var. scolymus]	Cynara cardunculus ...	64.7	64.7	100%	1e-13	100.00%	437	KVI00009.1
hypothetical protein Ccrd_012208 [Cynara cardunculus var. scolymus]	Cynara cardunculus ...	64.7	64.7	100%	1e-13	100.00%	389	KVI09405.1
elongation factor 1-alpha 1 isoform X2 [Lactuca sativa]	Lactuca sativa	64.7	64.7	100%	1e-13	100.00%	380	XP_042754064.1

Download GenPept Graphics

elongation factor 1-alpha [Helianthus annuus]

Sequence ID: XP_021981523.1 Length: 449 Number of Matches: 1

See 2 more title(s) See all Identical Proteins(IPG)

Range 1: 226 to 244 GenPept Graphics ▼ Next Match ▲ Previous Match

Score	Expect	Identities	Positives	Gaps
64.7 bits(145)	1e-13	19/19(100%)	19/19(100%)	0/19(0%)

```
Query  1    PKRPSDKPLRLPLQDVYKI  19
            PKRPSDKPLRLPLQDVYKI
Sbjct  226  PKRPSDKPLRLPLQDVYKI  244
```

Supplemental Figure s4. BLAST-P analysis results performed with the aminoacid sequence deduced from amplification product obtained with AtEF1-A primer pairs.

	AtAct2				***AtEF1-A***				***NtEF1-A***			
**Sample**	**Ct value**	**Experiment mean**	**SD**	**CV**	**Ct Value**	**Experiment mean**	**SD**	**CV**	**Ct Value**	**Experiment mean**	**SD**	**CV**
Shoot CTRL1	30,34	30,4866667	0,57422411	**0,05053975**	23,79	23,09	0,63624793	**0,02676146**	35,6	34,76	0,73430239	**0,02419668**
Shoot CTRL2	30				23,19				34,44			
Shoot CTRL3	31,12				22,29				34,24			
Shoot AsIII(6h)1	32,55	30,6366667	1,68953643		23,89	23,2333333	0,63610796		34,33	34,53	0,71140706	
Shoot AsIII(6h)2	30,01				23,19				35,32			
Shoot AsIII(6h)3	29,35				22,62				33,94			
Shoot AsIII(48h)1	26,16	27,6	1,29799846		22,62	23,2133333	0,61614392		33,74	33,9133333	0,52204725	
Shoot AsIII(48h)2	27,96				23,17				33,5			
Shoot AsIII(48h)3	28,68				23,85				34,5			
Shoot AsV(6h)1	30,54	29,3066667	1,06884673		22,79	23,6666667	0,94044316		34,89	34,8833333	1,38001208	
Shoot AsV(6h)2	28,65				23,55				36,26			
Shoot AsV(6h)3	28,73				24,66				33,5			
Shoot CdII(6h)1	30,1	29,5933333	0,83464563		23,03	22,9166667	0,36350149		33,72	34,1533333	0,93831409	
Shoot CdII(6h)2	28,63				22,51				33,51			
Shoot CdII(6h)3	30,05				23,21				35,23			
Root CTRL1	33,61	32,2733333	1,23313962	**0,04484553**	25,535	25,6616667	0,15892871	**0,03929649**	34,18	35,2266667	1,09258104	**0,03391399**
Root CTRL2	32,03				25,61				35,14			
Root CTRL3	31,18				25,84				36,36			
Root AsIII(6h)1	30,27	29,61	0,77948701		25,8	25,4666667	0,28884829		35,23	34,9266667	0,65957057	
Root AsIII(6h)2	29,81				25,31				35,38			
Root AsIII(6h)3	28,75				25,29				34,17			
Root AsIII(48h)1	30,21	30,3533333	1,4503218		26,57	25,8866667	0,78449559		35,21	34,3066667	0,8292366	
Root AsIII(48h)2	31,87				26,06				33,58			
Root AsIII(48h)3	28,98				25,03				34,13			
Root AsV(6h)1	31,78	31,73	0,836122		23,1	23,9033333	0,74096784		35,85	33,9033333	1,7350024	
Root AsV(6h)2	32,54				24,05				32,52			
Root AsV(6h)3	30,87				24,56				33,34			
Root CdII(6h)1	32,56	32,2633333	0,34151623		24,32	24,02	0,26457513		34,42	33,6566667	1,24500335	
Root CdII(6h)2	31,89				23,92				32,22			
Root CdII(6h)3	32,34				23,82				34,33			

Table S1. Validation of reference genes in *D. viscosa*. Raw Ct values of the three amplification products given by the primer pairs used to identify the reference genes for *Dittrichia* in all experimental condition and samples (shoots and roots). Means ± SD of single independent experiments and Coefficient of Variation (CV) are reported.
